# Comprehensive analysis of targetable oncogenic mutations in chinese cervical cancers

**DOI:** 10.18632/oncotarget.3212

**Published:** 2014-12-31

**Authors:** Libing Xiang, Jiajia Li, Wei Jiang, Xuxia Shen, Wentao Yang, Xiaohua Wu, Huijuan Yang

**Affiliations:** ^1^ Department of Gynecological Oncology, Fudan University Shanghai Cancer Center, Department of Oncology, Shanghai Medical College, Fudan University, Shanghai, China; ^2^ Department of Pathology, Fudan University Shanghai Cancer Center, Department of Oncology, Shanghai Medical College, Fudan University, Shanghai, China

**Keywords:** Oncogenic mutation, Cervical cancers, PI3K pathway genes, RTK genes, RAS genes

## Abstract

Mutations in 16 targetable oncogenic genes were examined using reverse transcription polymerase chain reaction (RT-PCR) and direct sequencing in 285 Chinese cervical cancers. Their clinicopathological relevance and prognostic significance was assessed. Ninety-two *nonsynonymous* somatic *mutations were identified in 29.8% of the cancers. The mutation rates were as follows: PIK3CA (12.3%), KRAS (5.3%), HER2* (*4.2%)*, *FGFR3-TACC3* fusions (3.9%), *PTEN* (2.8%), *FGFR2* (1.8%), *FGFR3* (0.7%), *NRAS* (0.7%)*, HRAS* (0.4%) and *EGFR* (0.4%). No mutations were detected in *AKT1* or *BRAF*, and the fusions *FGFR1-TACC1*, *EML4-ALK*, *CCDC6-RET* and *KIF5B-RET* were not found in any of the cancers*. RTK* and *RAS* mutations were more common in non-squamous carcinomas than in squamous carcinomas (P=0.043 and P=0.042, respectively). *RAS* mutations were more common in young patients (<45 years) (13.7% vs. 7.7%, P=0.027). *RTK* mutations tended to be more common in young patients, whereas *PIK3CA/PTEN/AKT* mutations tended to be more common in old patients. RAS mutations were significantly associated with disease relapse. To our knowledge, this is the first comprehensive analysis of major targetable oncogenic mutations in a large cohort of cervical cancer cases. Our data reveal that a considerable proportion of patients with cervical cancers harbor known druggable mutations and might benefit from targeted therapy.

## INTRODUCTION

Cervical cancer is the seventh most common and the eighth deadliest cancer in Chinese women. In 2010, it affected approximately 77,000 women and killed over 21,000 women in China [1]. Current treatment protocols for invasive cervical cancer are mostly based on surgery and chemoradiotherapy. Despite improved multidisciplinary treatment, the prognosis of advanced/recurrent cervical cancer is still poor, with a median overall survival (OS) ranging between 10 and 13 months [2].

A detailed understanding of somatic mutations in genes that encode signaling proteins with crucial roles in cellular proliferation and survival has led to the development of highly specific inhibitors that target key oncogenic pathways. Recently, molecularly targeted therapies have dramatically improved the treatment outcomes in patients whose tumors harbor activated mutant kinases such as mutant EGFR, BRAF and HER2 or translocated ALK [3-7]. The identification of such “druggable” mutant kinases in human cancer is becoming increasingly important. However, the question of whether cervical cancers harbor identifiable driver oncogenic mutations and benefit from targeted therapy has remained largely unanswered. Neither the prevalence of activating mutations nor the relevant clinicopathological characteristics are well established, and these factors are of paramount importance in the design of clinical trials for advanced or recurrent cervical cancers.

To investigate such “druggable” oncogenic genetic alterations in cervical cancer, we examined the mutational status of 16 oncogenic genes—*KRAS*, *NRAS*, *HRAS*, *BRAF*, *PIK3CA*, *PTEN*, *AKT1*, *HER2*, *EGFR*, *FGFR2* and *FGFR3* as well as *FGFR3-TACC3*, *FGFR1-TACC1*, *EML4-ALK*, *CCDC6-RET* and *KIF5B-RET* fusions—in a cohort of 285 Chinese patients with resected cervical cancer using reverse transcription polymerase chain reaction (RT-PCR) and direct sequencing.

## RESULTS

Tumors from 285 Chinese patients with cervical cancer were examined, including 179 patients with SCCs, 62 with ACs, 34 with ASCs, and 10 with other rare histopathological types. More extensive patient data are available in Supplementary Table S2.

### Mutation Profile

**Figure 1:**
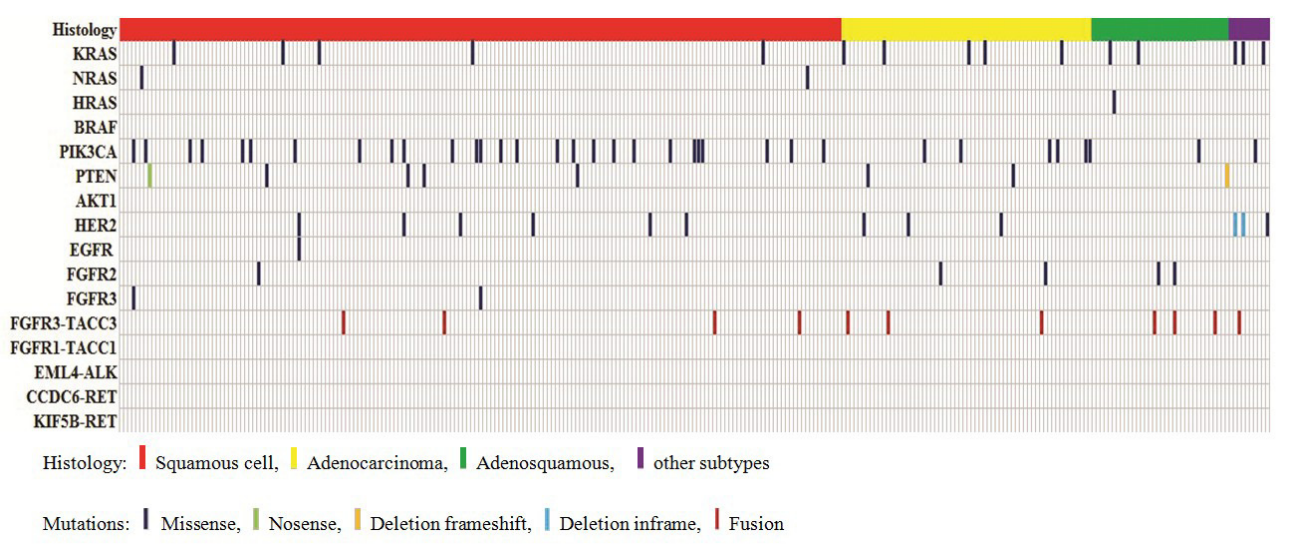
Distribution of mutations of the 16 tested genes in the 285 Chinese cervical cancers

A total of 92 nonsynonymous somatic mutations were identified in the 285 cervical cancers by Sanger sequencing, including 77 missense substitutions, 1 nonsense substitution, 2 in-frame deletions, 1 frameshift deletion and 11 in-frame fusions (Fig. 1, Supplementary Fig.S1 and Supplementary Table S3). The mutation rates of the tested genes were 27.4% (49 of 179) in SCC, 33.9% (21 of 62) in AC, 26.5% (9 of 34) in ASC and 60% (6 of 10) in the other rare histological subtypes. The mutation rates in AC and the other rare histological types were higher than that in SCC; however, these differences were not statistically significant (P=0.335 and P=0.066, respectively).

**Figure 2:**
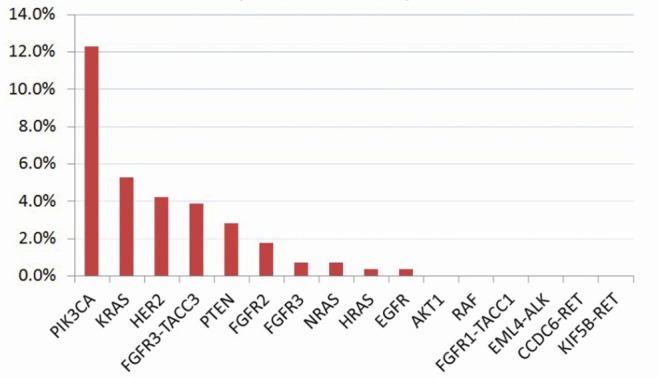
Mutation rates of the 16 tested genes in 285 Chinese cervical cancers

Eighteen (6.3%) cancers were found to harbor RAS missense mutations, including 15(5.3%) in *KRAS*, 2(0.70%) in *NRAS* and 1(0.4%) in *HRAS* (Fig. 2). Thirty-five (12.3%) cancers harbored *PIK3CA* mutations, including 32 occurring in exon 9 and 3 in exon 20. Among these mutations, E545K (c.1633G>A) and E542K (c.1624G>A) were found in 20 (7.0%) and 11 (3.8%) cancers, respectively; H1047R (c.3140A>G) was found in 2 cancers and was associated with an increased response to PI3K/AKT/mTOR signaling pathway inhibitors in a previous clinical trial [8]. Eight (2.8%) samples harbored *PTEN* somatic mutations. No mutations were found in *BRAF* or *AKT1*.

*HER2, EGFR, FGFR2* and *FGFR3* missense substitutions were observed in 10(3.5%), 1(0.4%), 5(1.8%) and 2(0.7%) of the cancers, respectively. Two small-cell neuroendocrine carcinomas harbored *HER2* in-frame deletions in exon 21.

**Figure 3:**
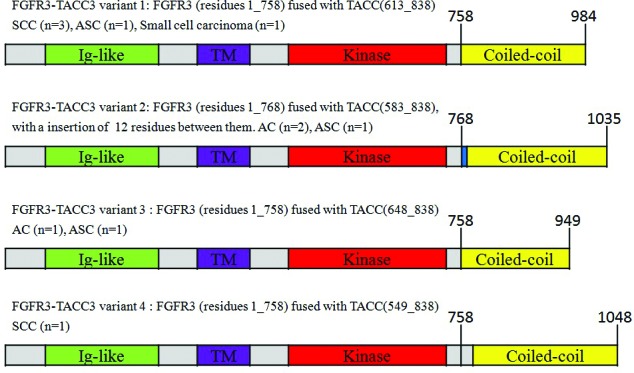
FGFR3-TACC3 fusion variants

Eleven (3.9%) cancers were found to harbor *FGFR3-TACC3* fusions. Four variants were identified (Fig. 3). The *FGFR1-TACC1, EML4-ALK, CCDC6-RET* and *KIF5B-RET* fusions were not found in any of the cancers.

### Clinicopathological characteristics of the patients with mutations

Table 1 shows the occurrence of the 16 oncogenic mutations in different groups according to different clinicopathological features. *RTK* mutations were more common in non-squamous carcinomas than in squamous carcinomas (15.1 vs. 7.3%, P=0.043). *RAS* mutations were also more frequent in non-squamous carcinomas than in squamous carcinomas (10.4 vs. 3.9%, P=0.042). *RAS* mutations were more common in young patients (<45 years) than in old patients (≥45 years)(13.7% vs. 7.7%, P=0.027). *RTK* mutations tended to be more common in young patients, whereas *PIK3CA/PTEN/AKT* mutations tended to be more common in old patients; however, these differences were not statistically significant. No correlation was found between the 16 oncogenic mutations and disease severity (deep stromal invasion, parametrial invasion, LVSI, lymph node metastasis and distant metastasis). Of the two patients exhibiting distant metastasis, one harbored a PIK3A mutation, whereas the other patient harbored a FGFR3-TACC3 fusion.

**Table 1:** The prevalence of the 16 oncogenic mutations in different groups according to different clinicopathological features

Characteristics	PIK3CA/PTEN/AKT			RTKs*			RAS/RAF		
	Wild-type	Mutation	P	Wild-type	mutation	P	Wild-type	mutation	P
Age(years)			0.065			0.114			**0.027**
＜45	105	12		101	16		105	12	
≥45	137	31		155	13		162	6	
Menopause status			0.078			0.209			0.441
Yes	73	19		86	6		88	4	
No	169	24		170	23		179	14	
Histological type			0.122			**0.043**			**0.042**
SCC	147	32		166	13		172	7	
AC+ASC+Others	95	11		90	16		95	11	
Tumor size			0.716			1.000			0.595
＞4cm	67	13		72	8		74	6	
≤4cm	175	30		184	21		193	12	
Depth of myometrial invasion			0.066			0.829			1.000
＞1/2	167	36		183	20		190	13	
≤1/2	75	7		73	9		77	5	
LVSI			1.000			0.209			0.604
Yes	78	14		86	6		85	7	
No	164	29		170	23		182	11	
Regional lymph node metastasis			0.194			0.658			1.000
Yes	61	15		67	9		71	5	
No	181	28		189	20		196	13	
Parametrial involvement			0.674			1.000			0.519
Yes	9	2		10	1		10	1	
No	233	41		246	28		257	17	
Distant metastasis			0.279			0.193			1.000
Yes	1	1		1	1		2	0	
No	241	42		255	28		265	18	

* RTKs included EGFR, HER2, FGFR2，FGFR3 mutations and FGFR3-TACC3 fusions. Fisher's exact test was employed to assess the difference between groups.

### Clinical outcome

**Figure 4:**
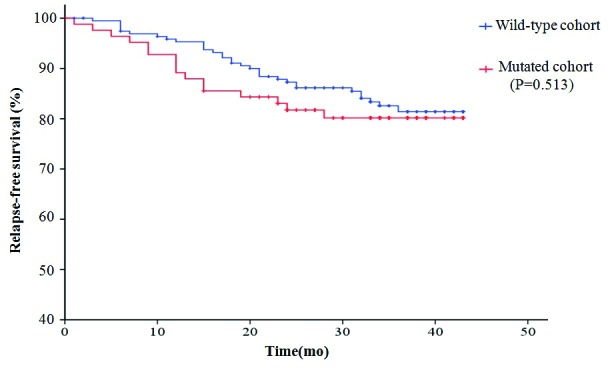

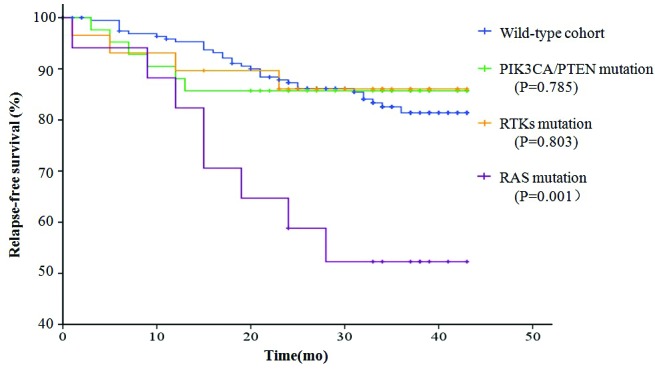
Disease-free survival (RFS) curves plotted by Kaplan-Meier method for the 285 patients based on the mutation status of the 16 tested genes A. Comparison of RFS in the patients with mutations and the patients without mutations. B. Comparison of RFS in patients with different mutations

Overall, 75.8% (216 of 285) of the patients received adjuvant therapies after surgery. The median follow-up duration was 35 months (from 20 to 43 months). During follow-up, recurrence information was available for 268 of 285 (94.0%) patients. Forty-nine patients experienced disease recurrence, including 16 patients with known oncogenic mutations. Because the follow-up data were not sufficiently mature, only RFS was assessed according to the mutation status of the tested genes. The 3-year RFS in patients with *RAS* mutations was 52.3%, which was significantly lower than that in patients with *PIK3CA/PTEN* mutations (85.7%), patients with *RTK* mutations (86.1%), and patients without mutations (81.4%). The disease-free survival in patients with mutated *PIK3CA/PTEN* or *RTK* was similar to that of patients without mutations (Fig. 4).

## DISCUSSION

Recently, the development of targeted therapeutics has made it both necessary and valuable to evaluate genetic alterations in various cancers and to subdivide tumors based on the results of molecular genotyping. Currently, drugs that target mutant *EGFR, RAF, HER2, ALK, RET* and *FGFRs* are clinically available [3-7, 9, 10]. To date, inhibitors of RAS, PIK3CA, PTEN or AKT1 have not been approved by US Food and Drug Administration (FDA); however, inhibitors of downstream targets such as mTOR and MEK have shown therapeutic efficacy in a wide variety of tumors [11-14]. Furthermore, small molecules that irreversibly bind to mutant KRAS (G12C) have been developed and have shown promising antitumor activity *in vitro* [15]. According to the Catalogue of Somatic Mutations in Cancer (COSMIC) database, the mutation profiles of these genes and their clinicopathological characteristics in cervical cancer are not well established. Oncogenic mutations were examined in samples that were very limited in size (typically less than 100 cases), and the gene mutation status was not concomitantly investigated [16-19]. In addition, it has remained unclear whether patients with cervical cancer harbor gene translocations. To the best of our knowledge, the present study is the first comprehensive and concurrent large-scale analysis of oncogenic mutations in Chinese patients with cervical cancer.

The main findings of this study were that 29.8% of the patients with cervical cancer harbored oncogenic mutations in the 16 genes assessed, and the mutation rates of *PIK3CA/PTEN*, *RTK* and *RAS* were 15.1%, 10.2%, and 6.3%, respectively. *RTK* and *RAS* mutations have been found to be more common in histological subtypes of non-SCCs that are resistant to radiation and chemotherapy and are associated with poor survival [20-22]. Approximately one third of the patients with recurrent disease harbored those targetable mutations. Thus, our data provide a clear rationale for clinical trials of targeted inhibitors in patients with cervical cancer especially those advanced and recurrent cases.

Another significant new finding is the discovery of *FGFR3-TACC3* fusions in cervical cancer. To our knowledge, this is the first report describing *FGFR3-TACC3* fusions in cervical cancer. We found that 3.9% of cervical cancers harbored these intrachromosomal translocations. The fusion proteins encoded by these genes transform normal cell lines, and FGFR kinase inhibitors counteract the oncogenic activity of these proteins [23-25]. Patients with tumors that carry *FGFR3-TACC3* fusions might potentially benefit from FGFR tyrosine kinase inhibitors, such as pazopanib, ponatinib, PD173074, AZD4547 and BGJ398 [26, 27].

In addition, our results confirm and extend previous findings reported in the literature. The *KRAS* mutation rate in this study (5.3%) was lower than that described in previous studies (6.3-13.9%) (19, 28, 29), which might be a result of the different distribution of histological subtypes or different populations. *BRAF* mutations in cervical cancer were not observed in this study, as previously reported in a study by Pappa et al. [28]. The frequency of *PIK3CA* mutations was 12.3% in this study, which was consistent with COSMIC data and the outcomes of a recent next-generation sequencing study [30]. In the present series of patients who underwent surgery-based multimodality therapy, patients harboring *PIK3CA* mutations did not have a survival disadvantage. However, McIntyre et al. reported that *PIK3CA* mutation was strongly associated with worse overall survival in early-stage cervical cancer patients treated with radical chemoradiotherapy [31]. Further investigations are warranted to confirm whether PI3K pathway inhibitors could improve radiotherapy efficacy in cervical cancer patients who have *PIK3CA* or *PTEN* mutations. Recurrent *HER2* mutations in cervical cancer were first discovered by Ojesina et al. [30], and our findings confirmed that *HER2* mutations were not unusual among cervical malignancies. *HER2* in-frame deletions in small-cell neuroendocrine carcinomas warranted further investigation and functional validation. Our study suggested that somatic mutations in *EGFR, FGFR2*, and *FGFR3* are rare in cervical cancers; this result was similar to the findings of previous studies [16, 17, 32].

Although many oncogenic gene mutations were analyzed in this study, there were some limitations. First, protein expression and gene copy number changes were not examined in this study, which might have prevented us from identifying patients who would be suitable for targeted therapy, such as those with *HER2* or *EGFR* overexpression. Second, not all mutations were located in well-known hotspots; thus, some of the mutations identified in this study might serve as ‘passenger mutations’. Third, the primers used for the translocation tests did not cover all isoforms of the tested genes. We could therefore not exclude the possibility that some gene fusions might have been missed.

In summary, our data reveal that a considerable proportion of patients with cervical cancers harbor known druggable mutations and might benefit from targeted therapy. These findings provide support for clinical trials involving PI3K pathway inhibitors, tyrosine kinase inhibitors (TKIs) and MAPK pathway inhibitors in patients with cervical cancer detected by targeted molecular screening.

## METHODS

### Patients and specimens

This research was approved by the Institutional Review Board of Fudan University Shanghai Cancer Center (FUSCC). All patients provided written informed consent. A total of 285 Chinese women were included in this study after it was determined that they met the following criteria: pathologically confirmed primary cervical squamous cell carcinoma (SCC), adenocarcinoma (AC), adenosquamous carcinoma (ASC) or small cell carcinoma; stage IB1-IIA2 disease according to the International Federation of Gynecology and Obstetrics (FIGO) staging system; no neoadjuvant chemotherapy or radiation; and no history of pre-operative cone biopsies. Cervical tumor specimens were collected during radical hysterectomy procedures from January 2011 to December 2012. Each specimen was cut into two blocks. One was fixed in formalin, embedded in paraffin, sectioned and stained with Hematoxylin & Eosin for histological examination. The other was stored in RNAlater solution (Ambion) at −80°C for mutational analysis. Only specimens in which tumor cells accounted for more than 50% of the whole tissue were processed to mutational analysis. Histopathological characteristics and tumor contents were confirmed by two pathologists (X Shen and W Yang). Clinical and pathological data were prospectively collected and included the following: age, menopause status, histological subtype, FIGO stage, tumor size, depth of invasion, lymphovascular space involvement (LVSI), lymph node status, distant metastasis, parametrial involvement and tumor differentiation. The patients were followed up in the clinic or by telephone to determine disease recurrence.

### Mutational analyses

RNA and genomic DNA were extracted from the tumor tissues according to the standard protocols provided with the DNA/RNA Isolation Kit (Tiangen Biotech); 2 μg of total RNA was reverse-transcribed into single-stranded cDNA using an M-MLV Reverse Transcriptase Kit(Invitrogen). The presence of mutations was determined in *NRAS* (exons 1-3), *KRAS* (all exons), *HRAS* (exons 1-3), *BRAF* (exons 13-16), *PIK3CA* (exons 8-11,18-20), *PTEN* (exons 5-9), *AKT1* (exons 1-3), *HER2* (exons 5-12, 16-25), *EGFR* (exons 18-22), *FGFR2*(exons 5-9,10-15) and *FGFR3* (exons 5-9) as well as in *FGFR3-TACC3*, *FGFR1-TACC1*, *EML4-ALK*, *CCDC6-RET* and *KIF5B-RET* fusions. cDNA was PCR amplified with KOD plus neo DNA polymerase (Toyobo). The PCR products were sequenced from both ends using Sanger sequencing. All mutations were confirmed by an additional independent PCR. The primers and the detailed PCR protocol are provided in Supplementary Table S1. Germline mutations were excluded by sequencing the DNA extracted from paired peripheral blood cells or corresponding formalin-fixed and paraffin-embedded normal uterus corpus tissues. Paraffin embedded tissues were retrieved from archival specimens stored in pathology department and reviewed by the two pathologists to exclude tumor involvements.

### Statistical analysis

The association between mutations and clinicopathological characteristics was analyzed by Fisher's exact test. Relapse-free survival (RFS) was calculated using the Kaplan–Meier method, and the differences between the groups were determined using the log-rank test. The 2-sided significance level was set at P<0.05. The data were analyzed using IBM SPSS Statistics 19 (IBM Inc.).

## SUPPLEMENTARY MATERIAL, FIGURE AND TABLES






