# Supplementary material for: Comprehensive analysis of targetable oncogenic mutations in chinese cervical cancers
Source: Oncotarget. 2014 Dec 31;6(7):4968–75. doi: 10.18632/oncotarget.3212 (PMC4467127; doi:10.18632/oncotarget.3212)
Supplement: Supplementary file 1 [file oncotarget-06-4968-s001.pdf]

Comprehensive analysis of targetable oncogenic mutations in chinese cervical cancers

Supplementary Material

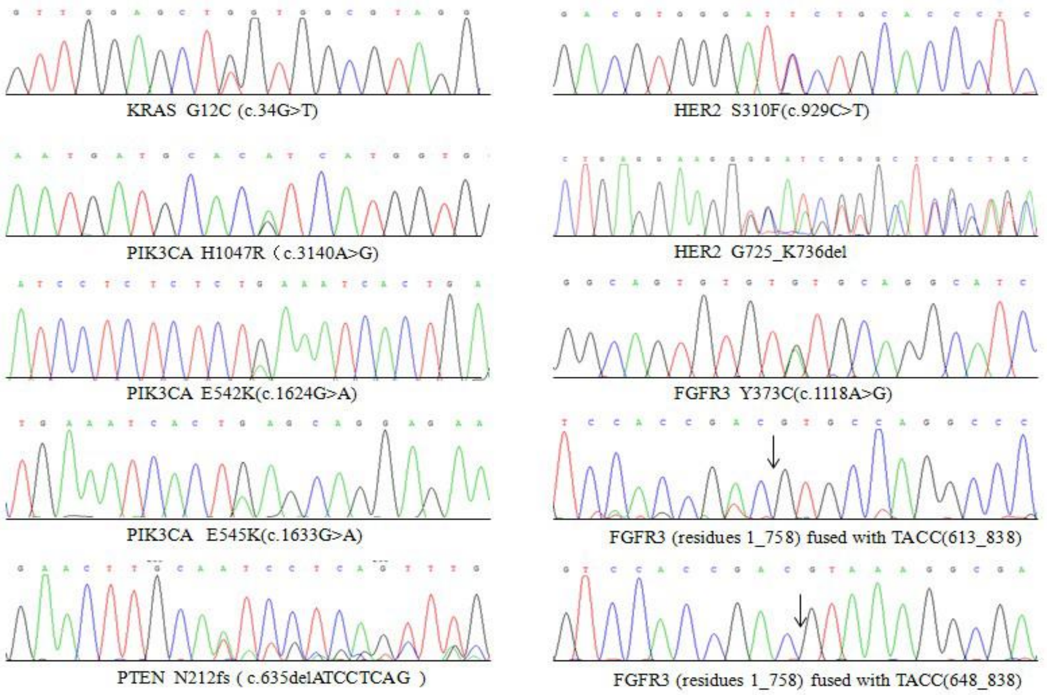

Supplementary Figure S1: Examples of the oncogenic mutations detected by reverse transcription polymerase chain reaction (RT-PCR) and direct sequencing in cervical cancers

Supplementary Table S2 Clinicopathological characteristics of the 285 patients with cervical cancer:

Variables		Cases (%)
Age（years）		
	＜45	117 (41.1)
	≥45	168 (58.9)
Menopause status		
	Yes	92 (32.3)
	No	193 (67.7)
Histological type		
	Squamous cell carcinomas	179 (62.8)
	Adenocarcinomas	62 (21.8)
	Adenosquamous carcinomas	34 (11.9)
	Others*	10 (3.5)
FIGO stage		
	IB1-IB2	145 (50.9)
	IIA1-IIA2**	140 (49.1)
Tumor size		
	＞4cm	80 (28.1)
	≤4cm	205 (71.9)
Depth of myometrial invasion		
	＞1/2	203 (71.2)
	≤1/2	82 (28.8)
LVSI		
	Yes	92 (32.3)
	No	193 (67.7)
Regional lymph node metastasis		
	Yes	76 (26.7)
	No	209 (73.3)
Parametrial involvement		
	Yes	11 (3.9)
	No	274 (96.1)
Distant metastasis		
	Yes	2 (0.7)
	No	283 (99.3)

*The histological subtypes of the 10 patients were neuroendocrine carcinomas (5), small cell carcinomas without evidence of neuroendocrine differentiation (3), primitive neurotodermal tumour (1) and adenosarcoma (1).

** Two patients was diagnosed with FIGO staged IIA diseases before surgery, but was found to have ovarian metastasis or abdominal wall metastasis during surgery.
